# Accelerated 3-Year MD Pathway Programs: Graduates’ Perspectives on Education Quality, the Learning Environment, Residency Readiness, Debt, Burnout, and Career Plans

**DOI:** 10.1097/ACM.0000000000004332

**Published:** 2022-01-26

**Authors:** Shou Ling Leong, Colleen Gillespie, Betsy Jones, Tonya Fancher, Catherine L. Coe, Lisa Dodson, Matthew Hunsaker, Britta M. Thompson, Angela Dempsey, Robert Pallay, William Crump, Joan Cangiarella

**Affiliations:** 1 **S.L. Leong** is assistant dean, Pathways Innovation, and director, 3+ Accelerated Pathway, Department of Family and Community Medicine, Penn State College of Medicine, Hershey, Pennsylvania; ORCID: http://orcid.org/0000-0003-2954-5381.; 2 **C. Gillespie** is director, Division of Education Quality, Institute for Innovations in Medical Education, New York University Grossman School of Medicine, New York, New York.; 3 **B. Jones** is chair, Department of Medical Education, and codirector, Family Medicine Accelerated Track, Texas Tech University Health Sciences Center School of Medicine, Lubbock, Texas.; 4 **T. Fancher** is associate dean, Workforce Innovation and Community Engagement, University of California Davis School of Medicine, Sacramento, California.; 5 **C.L. Coe** is assistant professor of family medicine and director, Fully Integrated Readiness for Service Training (FIRST) Program, University of North Carolina at Chapel Hill School of Medicine, Chapel Hill, North Carolina.; 6 **L. Dodson** is campus dean, Medical College of Wisconsin–Central Wisconsin, Wasau, Wisconsin.; 7 **M. Hunsaker** is campus dean, Medical College of Wisconsin–Green Bay, Green Bay, Wisconsin.; 8 **B.M. Thompson** is associate dean, Assessment and Evaluation, Penn State College of Medicine, Hershey, Pennsylvania.; 9 **A. Dempsey** is associate dean, Curriculum in the Clinical Sciences, Medical University of South Carolina College of Medicine, Charleston, South Carolina.; 10 **R. Pallay** is chair and program director, Family Medicine, Mercer University School of Medicine, Macon, Georgia.; 11 **W. Crump** is associate dean, Trover Campus, University of Louisville School of Medicine, Madisonville, Kentucky.; 12 **J. Cangiarella** is associate dean, Education, Faculty and Academic Affairs, and director, Accelerated Three Year MD Pathway, New York University Grossman School of Medicine, New York, New York.

## Abstract

**Purpose:**

To compare perception of accelerated and traditional medical students, with respect to satisfaction with education quality, and the learning environment, residency readiness, burnout, debt, and career plans.

**Method:**

Customized 2017 and 2018 Medical School Graduation Questionnaires (GQs) were analyzed using independent samples *t* tests for means and chi-square tests for percentages, comparing responses of accelerated MD program graduates (accelerated pathway [AP] students) from 9 schools with those of non-AP graduates from the same 9 schools and non-AP graduates from all surveyed schools.

**Results:**

GQ completion rates for the 90 AP students, 2,573 non-AP students from AP schools, and 38,116 non-AP students from all schools in 2017 and 2018 were 74.4%, 82.3%, and 83.3%, respectively. AP students were as satisfied with the quality of their education and felt as prepared for residency as non-AP students. AP students reported a more positive learning climate than non-AP students from AP schools and from all schools as measured by the student–faculty interaction (15.9 vs 14.4 and 14.3, respectively; *P* < .001 for both pairwise comparisons) and emotional climate (10.7 vs 9.6 and 9.6, respectively; *P* = .004 and .003, respectively) scales. AP students had less debt than non-AP students (*P* < .001), and more planned to care for underserved populations and practice family medicine than non-AP students from AP schools (55.7% vs 33.9% and 37.7% vs 9.4%; *P* = .002 and < .001, respectively). Family expectations were a more common influence on career plans for AP students than for non-AP students from AP schools and from all schools (26.2% vs 11.3% and 11.7%, respectively; *P* < .001 for both pairwise comparisons).

**Conclusions:**

These findings support accelerated programs as a potentially important intervention to address workforce shortages and rising student debt without negative impacts on student perception of burnout, education quality, or residency preparedness.

The 2010 Carnegie report recommended that medical education become more individualized and include the option of accelerating training. ^1^ Furthermore, some have argued that there is substantial waste in medical education and that training can be shortened by 30% without compromising physician competence. ^2^ Accelerated medical school programs allow students to obtain their medical degree in 3 years. ^3,4^ By graduating students to enter the workforce 1 year earlier than traditional programs, accelerated programs can help address the continuing physician shortage ^5^ (projected to rise to between 46,900 and 121,900 by 2032) ^6^ and rising medical school debt (median of $200,000). ^7^

For decades, McMaster University and the University of Calgary, in Canada, have been awarding MD degrees after 3 years. ^8^ Compared with graduates of traditional 4-year programs in the United States and Canada, 3-year MD graduates were found to have performed similarly on standardized national examinations, during residency, and when competing for their preferred residency positions. ^8^ A 2014 survey found that 35% of U.S. medical schools have or are considering offering the option of 3-year MD pathways. ^9^ The Consortium of Accelerated Medical Pathway Programs (CAMPP), funded by the Josiah Macy Jr. Foundation, started in 2015 with 8 U.S. and Canadian medical schools and as of 2017, had tripled its membership. ^3^ CAMPP member schools have diverse missions ranging from a pathway focused on training primary care physicians to practice in rural or underserved communities to entire 3-year MD campuses or schools. ^10–13^ Details of these programs, including their curricula, missions, specialty focus, and size, have been described elsewhere by Cangiarella et al ^3^ and Leong et al. ^4^ Some accelerated programs offer graduates direct progression into residency in the home school through the National Resident Matching Program (NRMP), seamlessly linking undergraduate and graduate medical education and continuity of patient care, learning, assessment, and coaching.

Some have raised concerns that accelerated programs compromise student competency and readiness for residency (e.g., level of independence, depth of clinical experience and exposure, level of maturity and responsibility). ^9,14^ Skeptics argue that the growing complexity of medical knowledge may make it counterproductive to compress the curriculum into 3 years and that such compression has the potential to add to student stress and burnout.

Because most 3-year MD programs are too new to measure residency or postresidency outcomes, student reports of their experience and outcomes are important early measures for assessing the success of such programs. To better understand the experience of accelerated 3-year MD students, we used customized Association of American Medical Colleges (AAMC) Medical School Graduation Questionnaire (GQ) reports to compare the perception of accelerated and traditional students, with respect to the following aspects of the student experience: satisfaction with the quality of their education, the learning environment, readiness for residency, burnout, debt, and career plans.

## Method

The GQ is a national questionnaire administered annually by the AAMC to medical students in their final year at all U.S. medical schools. ^15^ The survey is designed for medical schools to use in program evaluation and to improve the medical student experience and includes questions related to preclinical, clinical, and elective experiences; general medical education and readiness for residency; student services; experiences of negative behaviors; financial aid and indebtedness; career intentions; burnout; and the strengths of and areas that need improvement in the medical school. While the AAMC uses students’ American Medical College Application Service identifiers in the GQ, the student data used in this report are deidentified, reported in aggregate, and not broken out by participating schools or programs.

We requested and received from the AAMC customized GQ aggregate reports for 3 groups of graduates from the classes of 2017 and 2018. ^16^ The first group included the 3-year MD students from 9 accelerated MD programs: New York University Grossman School of Medicine, Penn State College of Medicine, Texas Tech University Health Sciences Center School of Medicine, University of California Davis School of Medicine, University of North Carolina at Chapel Hill School of Medicine, Medical College of Wisconsin, Medical University of South Carolina, College of Medicine, Mercer University School of Medicine, and University of Louisville School of Medicine. Students enrolled in these accelerated programs were denoted as accelerated pathway (AP) students. The second group included traditional, nonaccelerated graduates from these same 9 schools, designated non-AP students from AP schools. This group allowed us to compare AP students with non-AP students within the same schools. The third group included traditional, nonaccelerated graduates from all U.S. medical schools surveyed in 2017 and 2018, designated non-AP students from all schools. The GQ had 15,609 responses from 140 schools in 2017 and 16,223 responses from 141 schools in 2018. ^15^ Using these national data, we sought to explore whether there were differences in domains (see below) relevant to the goals and structure of AP programs between graduates of AP programs and graduates of traditional 4-year (i.e., non-AP) programs both at the same schools and across all medical schools. We focused on students’ report of the following domains: satisfaction with their medical school experience, including the quality of their education, the learning environment, burnout, readiness for residency, debt, and career plans. We hypothesized that AP students would report lower levels of debt and greater intention to care for vulnerable populations and to practice family medicine but did not have a strong evidence base for predicting whether AP students would differ in their satisfaction with medical school, burnout, or sense of preparedness for residency.

### Analyses

Responses to the selected questions were combined across the 2017 and 2018 GQs. Response rates varied by question and are therefore included in each cell in the tables. Where response options were based on the percentage of students choosing from a 5-point disagree/agree scale, we calculated top box ratings by combining responses to the top categories (i.e., responses of strongly agree and agree) and then used chi-square tests to compare the percentage of AP students with those of non-AP students from AP schools and non-AP students from all schools (2 separate group comparisons). Mean reported levels of burnout and the learning environment were similarly compared using independent samples *t* tests (calculated based on the reported means, standard deviations, and sample sizes). We also compared AP students with the non-AP groups (same schools and all schools) in terms of background and sociodemographic characteristics, debt levels, and career plans, using chi-square tests to compare distributions across relevant categories. Because the data were aggregated across schools, we calculated statistics for each comparison independently and therefore sought to control for experiment-wise error by setting our significance level as *P* < .001, which balanced our need to avoid type I error with the small sample size of AP students. We were not able to conduct additional analyses (e.g., multivariate analyses, subgroup analyses) with the available data because only aggregate data were available.

### Measures

In terms of specific measures, the GQ uses a shortened 7-item version of the Medical School Learning Environment Survey instrument to assess the learning environment via 2 scales: emotional climate and student–faculty interaction. Emotional climate is measured by 3 items, with a total possible range of 0–15, and student–faculty interaction is measured by 4 items, with a total possible range of 0–20. Higher scores suggest a more positive learning environment. The GQ also includes the Oldenburg Burnout Inventory for Medical Students instrument which consists of 16 items on the exhaustion and disengagement scales. Each of these scales includes 8 items, with a total possible range of 0–24. Higher scores signify higher burnout.

The New York University Institutional Review Board approved this study (study #: s16-02152).

## Results

In 2017 and 2018, a total of 90 students graduated from the 9 U.S. schools with 3-year MD pathways included in our study. Across these 2 years, 67/90 (74.4%) AP students completed the GQ. In the same years, 2,117/2,573 (82.3%) non-AP students from AP schools and 31,765/38,116 (83.3%) non-AP students from all schools completed the GQ.

### Background and sociodemographic characteristics

There were no significant differences between AP students and either group of non-AP students in terms of gender, age, race/ethnicity, marital status, or having dependents (Table 1).

**Table 1:**
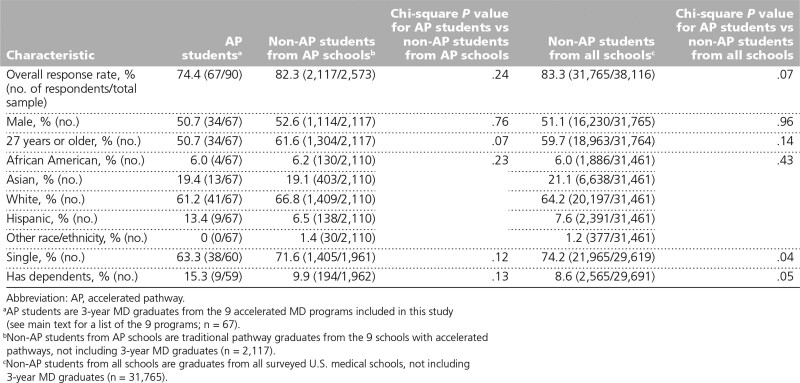
Background and Sociodemographic Characteristics of AP Students, Traditional Pathway Students From AP Schools, and Traditional Pathway Students From All Schools, Based on Responses to the 2017 and 2018 Association of American Medical Colleges Medical School Graduation Questionnaires

### Satisfaction with education quality

The majority of students in all 3 groups agreed or strongly agreed that they were satisfied with the quality of their medical education (Table 2), with slightly more AP students (65/67, 97.0%) reporting being satisfied than either of the non-AP student groups, although the differences did not meet our threshold for statistical significance. Substantially more AP students (59/67, 88.1%) reported agreeing that their basic science coursework was sufficiently clinically relevant than their non-AP peers at the same schools (1,601/2,080, 77.0%) and at all schools (24,786/31,340, 79.1%) and that their medical school had “done a good job of fostering and nurturing [their] development as a person” (AP students: 53/60, 88.3%; non-AP students from AP schools: 1,426/1,940, 73.5%; non-AP students from all schools: 21,357/29,275, 73.0%).

**Table 2:**
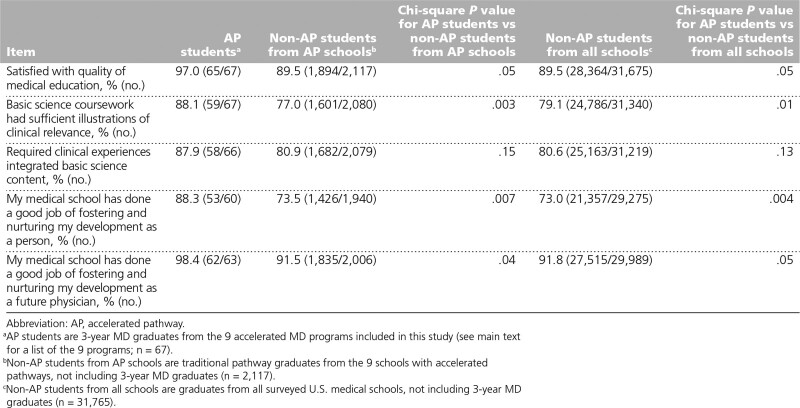
Satisfaction With Education Measures (Agree or Strongly Agree Responses) of AP Students, Traditional Pathway Students From AP Schools, and Traditional Pathway Students From All Schools, Based on Responses to the 2017 and 2018 Association of American Medical Colleges Medical School Graduation Questionnaires

### Learning environment and burnout

AP students rated the emotional climate and student–faculty interaction of the learning environment more positively than non-AP students from AP schools and non-AP students from all schools (Table 3). The analyses for student–faculty interaction for AP students, non-AP students from AP schools, and non-AP students from all schools (15.9 vs 14.4 and 14.3, respectively) were statistically significant (*P* < .001 for both pairwise comparisons), with the analyses for emotional climate (10.7 vs 9.6 and 9.6, respectively) approaching but not reaching statistical significance (*P* = .004 for AP students vs non-AP students from AP schools and *P* = .003 for AP students vs non-AP students from all schools). However, in terms of burnout, the mean scores on the exhaustion subscale were very similar across all 3 groups and, although AP students reported a lower mean score for the disengagement scale, it was not significantly lower than either of the other groups’ mean scores.

**Table 3:**
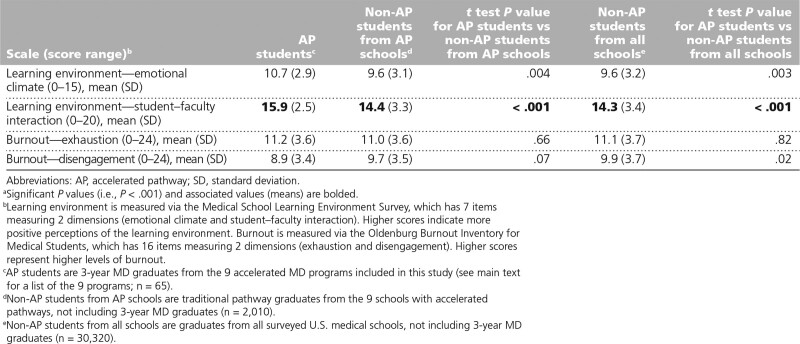
Learning Environment and Burnout Measures of AP Students, Traditional Pathway Students From AP Schools, and Traditional Pathway Students From All Schools, Based on Responses to the 2017 and 2018 Association of American Medical Colleges Medical School Graduation Questionnaires^a^

### Preparedness for residency

In the analyses of students’ perceptions of their preparedness for residency (Table 4), we found that AP students reported feeling as prepared for residency as their non-AP colleagues from AP schools and from all schools, with the majority of AP students agreeing or strongly agreeing that they were prepared for residency across the 7 preparedness items in the GQ (responses ranged from 61/65, 93.8% to 64/65, 98.5%).

**Table 4:**
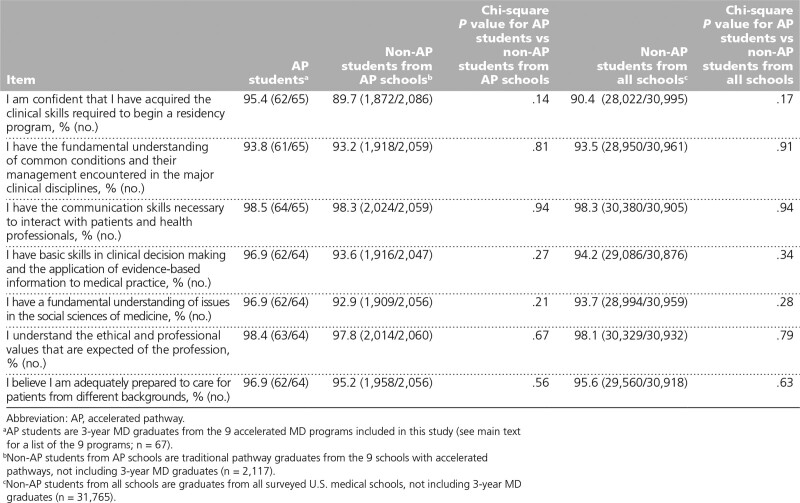
Perceived Preparedness for Residency Measures (Agree or Strongly Agree Responses) of AP Students, Traditional Pathway Students From AP Schools, and Traditional Pathway Students From All Schools, Based on Responses to the 2017 and 2018 Association of American Medical Colleges Medical School Graduation Questionnaires

### Debt

Analysis of the loan burden of students indicated that 39/58 (67.2%) AP students had less than $100,000 in loans at the end of medical school (including those with no debt), while only 743/1,914 (38.8%) non-AP students from AP schools and 11,379/28,954 (39.3%) non-AP students from all schools reported graduating with less than $100,000 in loans, a statistically significant difference for both pairwise comparisons (*P* < .001; Table 5). More AP students reported no medical school debt compared with the other groups (AP students: 24/58, 41.4%; non-AP students from AP schools: 537/1,914, 28.1%; non-AP students from all schools: 8,096/28,954, 28.0%; *P* < .001 for both pairwise comparisons), and a smaller percentage reported having debt equal to or greater than $150,000 (AP students: 8/58, 13.8%; non-AP students from AP schools: 960/1,914, 50.2%; non-AP students from all schools: 14,525/28,954, 50.2%; *P* < .001 for both pairwise comparisons). The percentage of students without debt before medical school was similar (AP students: 40/58, 69.0%; non-AP students from AP schools: 1,303/1,914, 68.1%; non-AP students from all schools: 19,544/28,954, 67.5%; data not presented in a table). (While New York University Grossman School of Medicine announced in August 2018 that the medical school would be tuition free moving forward, this had no impact for the students in this study who graduated in 2017 and 2018.)

**Table 5:**
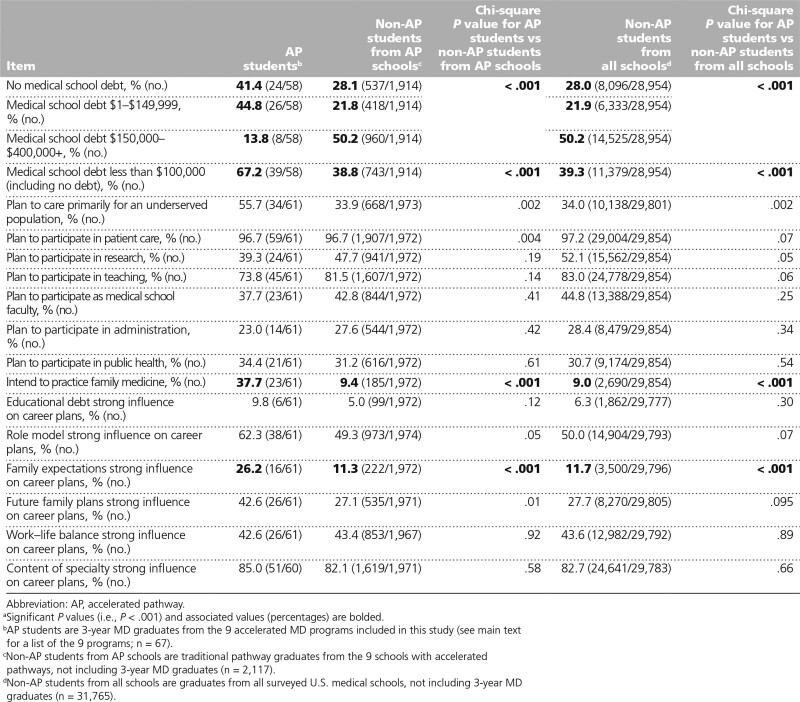
Debt and Career Plan Measures of AP Students, Traditional Pathway Students From AP Schools, and Traditional Pathway Students From All Schools, Based on Responses to the 2017 and 2018 Association of American Medical Colleges Medical School Graduation Questionnaires^a^

### Career plans

More AP students planned to care for an underserved population than either group of non-AP students (AP students: 34/61, 55.7%; non-AP students from AP schools: 668/1,973, 33.9%; non-AP students from all schools: 10,138/29,801, 34.0%; Table 5), though these differences were not statistically significant (*P* = .002 for both pairwise comparisons). A statistically significant greater percentage of AP students intended to practice family medicine (AP students: 23/61, 37.7%; non-AP students from AP schools: 185/1,972, 9.4%; non-AP students from all schools: 2,690/29,854, 9.0%; *P* < .001 for both pairwise comparisons). Factors that influenced career plans were similar among the 3 groups with the exception that more AP students reported that family expectations and future family plans were a strong influence on their career plans, and the analyses for family expectation were statistically significant (AP students: 16/61, 26.2%; non-AP students from AP schools: 222/1,972, 11.3%; non-AP students from all schools: 3,500/29,796, 11.7%; *P* < .001 for both pairwise comparisons).

## Discussion

The accelerated MD graduates in our study reported being more likely to enter family medicine and to care for medically underserved populations. These accelerated students also reported feeling prepared for residency with no adverse impact on their burnout levels. Additionally, they reported that their medical school education was of high quality and that they were graduating with less student debt.

The last decade has witnessed the growth of accelerated 3-year MD programs, ^9,17^ with CAMPP tripling its membership since 2015 and continuing to welcome new programs. However, these programs are not new. In the 1970s, 25% of U.S. medical schools offered 3-year programs. ^17,18^ These programs flourished in part due to federal financial support based on the premise that they could help address the looming physician shortage. ^17–20^ Despite data that showed equal performance among graduates of 3-year and 4-year programs, decreasing concern for physician shortages and the loss of federal funding led most of these 3-year programs to close. ^18^ In the 1990s, some 3-year programs (25) used a model whereby the final year of medical school overlapped with the first year of family medicine or primary care residency. ^17,20^ Most of these programs later closed due to accreditation concerns over learners functioning as postgraduate year 1 residents without their terminal medical degree. ^17^

Studies of the accelerated programs in the 1970s showed no major differences in the performance of graduates of 3-year and 4-year programs. ^21–23^ However, concerns that accelerated program graduates may be less competent and lack skills needed for residency remained. ^14^ Residency program directors have also remained apprehensive about accepting accelerated students. ^9^ In a recent survey, only 34% of residency program directors said they would accept accelerated students from schools outside of their own institution. ^9^ Our study suggests that these fears may not be justified as our cohorts of accelerated students reporting feeling as prepared for residency as nonaccelerated students.

With more medical schools developing innovative and efficient curricula to reduce the cost of medical education, it is important to maintain high-quality education and ensure the competence of the workforce. According to our study, graduates from accelerated 3-year MD programs feel as prepared for residency and as satisfied with their medical education as their nonaccelerated peers. Contributing factors to these findings may include curricular innovations, such as early clinical exposure, longitudinal mentoring and coaching, and experiences tailored to the accelerated experience.

Quality of life issues, including the intense pace of training, were also raised as part of the reason for accelerated programs failing in the 1970s and 1980s. ^19^ Our study found no significant differences in Oldenburg Burnout Inventory for Medical Students scores between accelerated and nonaccelerated students, suggesting that burnout may not be more common for accelerated students. Given that many accelerated 3-year MD pathways were designed to prepare medical students to enter partner residency programs through the NRMP or an NRMP all-in exception, students may well be relieved of some Match-related stressors that affect their peers in traditional programs. ^24^ Additionally, students in accelerated programs are generally integrated into a specific department and intended specialty earlier than their nonaccelerated counterparts, and the close mentorship, personal engagement, and autonomy that may result from this earlier integration may help mitigate burnout. ^25^ In addition, many of the AP programs have made pedagogical modifications in their curricula, such as reducing the number of electives to achieve graduation competencies to avoid overcrowding the curriculum. To reduce stress and potential burnout, AP programs have incorporated mentoring and coaching programs to help students to reduce stress and manage demands. ^26^ We saw possible evidence of these influences in our accelerated students’ reporting of more positive learning environments for the student–faculty interaction dimension.

In this study, students in accelerated programs reported having lower debt levels, which is generally the main goal of accelerated programs. The degree of debt relief will differ based on the school offering the accelerated program, especially given the diversity of accelerated program schools and their varying costs to students in terms of tuition, fees, and living expenses. Some accelerated program schools also have scholarship or tuition forgiveness in addition to requiring 1 less year of tuition. Moreover, with the rising costs of applying, interviewing, and (prepandemic) travel for residency interviews through the NRMP Match, some accelerated students receive additional cost savings due to a program’s direct progression into a partner residency. Additionally, the financial benefit of entering the workforce 1 year earlier is significant not only in terms of earning a full salary a year earlier but also in terms of the ability to pay back loans earlier. ^20^ As we work toward a diverse workforce, lowering the cost of medical education would add to efforts to make medical education more accessible and affordable to students from lower socioeconomic backgrounds; accelerated programs may be one way to achieve this.

This study has several limitations. Due to the limited number of accelerated programs with available data at the time of this study, the sample size of accelerated students is small and the programs they are from vary in their missions, curricula, size, and whether they offer direct progression into a partner residency. For example, with the exceptions of New York University Grossman School of Medicine, Texas Tech University Health Sciences Center School of Medicine, and Medical College of Wisconsin, the accelerated programs in our study graduated small numbers of students in 2017 and 2018 (i.e., 2–6 per year). In addition, the aggregate data necessitated multiple statistical tests, leading us to choose a conservative significance level to identify statistically significant differences. Data were self-reported and aggregated, limiting our ability to conduct more sophisticated analyses that could control for individual differences or analyze results by specific subgroups. There may be a ceiling effect associated with the preparedness for residency, as most respondents felt very prepared. Finally, we cannot separate out selection effects (e.g., who applies and who is admitted to the accelerated program) from curriculum effects (e.g., the impact of the accelerated programs). A goal of CAMPP is to pool data across schools so as to follow a large cohort of students longitudinally across medical school, residency, and into practice; this should be explored in future work. Future research should also explore in greater depth the experiences of accelerated students and views of residency program directors through interviews and questionnaires.

Perhaps most noteworthy about our findings is how few significant differences there were between the groups other than expected differences in debt levels and future practice intentions. We did find some evidence suggesting that the accelerated programs are perceived as more effective by students. Thus, our findings support accelerated programs as a potentially important intervention to address workforce shortages and rising student debt without negative impacts on student perception of burnout, education quality, or preparedness for residency. Future efforts should focus on the unique contributions of accelerated programs as these may show great promise for reengineering elements of medical education, including in nonaccelerated programs, to better align training to the health care needs of the nation and therefore, ultimately, may benefit both students and the public.

## Acknowledgments:

The authors wish to acknowledge Joy Bowen for her assistance with the preparation of this article and the Association of American Medical Colleges (AAMC) for the creation of the customized Medical School Graduation Questionnaire (GQ) reports.
